# Second opinion for degenerative spinal conditions: an option or a necessity? A prospective observational study

**DOI:** 10.1186/s12891-017-1712-0

**Published:** 2017-08-17

**Authors:** Mario Lenza, Rachelle Buchbinder, Margaret P. Staples, Oscar F.P. dos Santos, Reynaldo A. Brandt, Claudio L. Lottenberg, Miguel Cendoroglo, Mario Ferretti

**Affiliations:** 1 Hospital Israelita Albert Einstein, Avenida Albert Einstein, 627/701 – Jardim Leonor – CEP, São Paulo, SP 05652-900 Brazil; 2 Monash Department of Clinical Epidemiology, Cabrini Institute, Monash University, 181-183 Wattletree Rd, Malvern VIC, Melbourne, 3144 Australia; 3 Department of Epidemiology and Preventive Medicine, School of Public Health and Preventive Medicine, Monash University, 181-183 Wattletree Rd, Malvern VIC, Melbourne, 3144 Australia; 4 Avenida Albert Einstein, 627/701, bloco A1 – Programa Locomotor – Jardim Leonor – CEP, São Paulo, SP 05652-900 Brazil

**Keywords:** Spine [MeSH], Back pain [MeSH], Surgical procedures, Operative [MeSH], Referral and consultation [MeSH], Unnecessary procedures [MeSH]

## Abstract

**Background:**

Second opinions may improve quality of patient care. The primary objective of this study was to determine the concordance between first and second diagnoses and opinions regarding need for spinal surgery among patients with back or neck pain that have been recommended spinal surgery.

**Methods:**

We performed a prospective observational study of patients who had been recommended for spinal surgery and received a second opinion between May 2011 and May 2012 at the Hospital Israelita Albert Einstein on the advice of their health insurance company. A physiatrist and orthopaedic surgeon independently performed the second assessment. If both agreed surgery was indicated, or consensus could not be reached, participants attended a spine review panel for a final recommendation. Descriptive analyses compared diagnoses and management plans of the first and second opinions.

**Results:**

Of 544 referred patients, 16 (2.9%) did not meet inclusion criteria, 43 (7.9%) refused participation and 485 were included. Diagnoses differed from the first opinion for 290 (59.8%). Diagnoses of cervical and lumbar radiculopathy were concordant in 36/99 (36.4%) and 116/234 (49.6%) respectively. The second opinion was for conservative treatment for 168 (34.6%) participants, 27 (5.6%) were not considered to have a spine condition, and 290 (59.8%) were referred to the review board. 60 participants did not attend the board review and therefore did not receive a final recommendation. Board review was conservative treatment for an additional 67 participants, 20 were not considered to have a spine condition and 143 participants were recommended surgery. Overall, 33.6% received a final opinion of surgery (143/425) although only 66 (15.5%) received the same surgical recommendation, 235 (55.3%) were advised to have conservative treatment, and 47 (11.1%) were not considered to have a spinal diagnosis.

**Conclusions:**

We found a large discordance between first and second opinions regarding diagnosis and need for spinal surgery. This suggests that obtaining a second opinion could reduce potentially unnecessary surgery.

**Trial registration:**

Current Controlled Trials ISRCTN07143259. Registered 21 November 2011.

## Background

According to the latest Global Burden of Disease study, back pain is the most common cause of disability worldwide [1]. In the United States the estimated costs related to the population with spine complaints increased by 65% from 1997 to 2005; faster than overall health expenditures [2]. While the majority of people with non-specific back pain recover or have a recurrent course, a small group have persistent pain and/or radicular symptoms and a proportion of these are offered surgery [3, 4].

The costs of surgery for spinal disorders have increased over the last two decades. Costs of spine fusion have increased exponentially, and they are associated with the highest aggregate costs (relating to implants and hospitalisation) [5, 6]. Advances in healthcare technology, more sensitive diagnostic tools and the ageing population may be partially responsible for the increasing number of spinal surgeries. However wide practice variations in spine procedure rates also suggests overuse as a factor [7, 8].

One way of reducing potentially unnecessary surgery is to require a second opinion. Second opinions have been used as a tool to improve the quality of patients’ care in all private care systems, and are most likely to be requested when surgery is advised [9, 10]. While guidelines consistently recommend obtaining a second opinion when spinal surgery is advised [11–13], there is currently a paucity of published evidence confirming its effectiveness.

Brazil is a large country with wide social disparities. The health care system comprises combined private and public run programs with three subsectors: the public – financed by the state; the private (for-profit and non-profit) – financed by public or private funds; and private health insurance – with different forms of private health plans [14]. Only 24.7% of the population is covered by private health insurance. In the private health insurance care system in Brazil, when a spine surgeon recommends surgery for a patient, the insurance company usually requests a second opinion and the patient may also request one. In either case it is not mandatory and patients are reimbursed for received treatment irrespective of whether or not they agree to a second opinion and/or proceed with surgery despite a differing second opinion.

The primary aim of this prospective observational study was to compare the diagnoses and recommended management of patients with private health insurance who had received an initial recommendation for spinal surgery from a community-based spinal surgeon, and who were offered a second opinion by their private health insurers at the Hospital Israelita Albert Einstein (HIAE), a private not-for-profit philanthropic hospital. The outcome of the second opinion is required within 21 days. Our secondary aim was to compare functional and quality of life endpoints in a subset of patients who were subsequently treated at HIAE with either surgery or conservative care.

## Methods

All patients referred to the Spine Centre of HIAE for a second opinion between May 2011 and May 2012 were offered study participation if they met the following criteria: initial recommendation for spine surgery from a spine surgeon not affiliated with HIAE, adults over 18 years with no medical contraindication to general anaesthesia, an understanding of Portuguese and written informed consent. People with spinal fractures, major scoliosis, congenital spinal deformity, spondyloarthropathies, spinal tumours or infection were excluded. The Institutional Review Board approved the study (number 1592–12).

### Setting

HIAE is organised as a hub and spokes healthcare system, with a high complexity 640-bed hospital and five satellite units. The orthopaedic department comprises an inpatient unit and is organised as a service line. Physicians are typically self-employed and not hired by the hospital, except for the emergency department, diagnostic units and programs such as the Spine Centre. Within the Spine Centre, there are five physiatrists and three general orthopaedic surgeons, all employed by HIAE. They are responsible for performing second opinion assessments. All of them have expertise in the diagnosis and management of people with neck and back problems and all have had at least five years experience in this role.

### Procedure

**Fig. 1:**
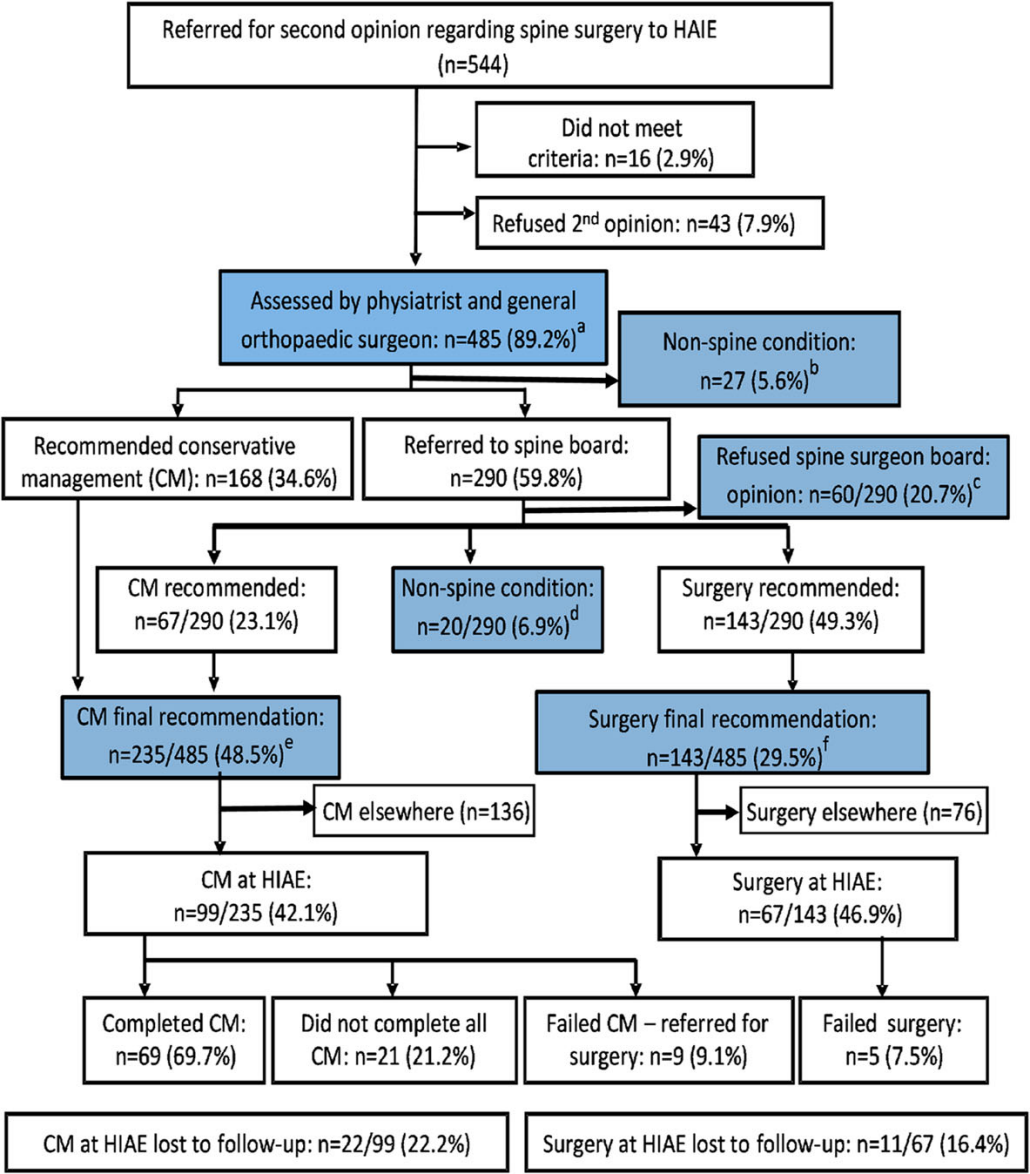
Second opinion flow chart. The boxes highlighted in blue summarise the main results: of the 425 participants who consented, were eligible to participate and agreed to complete the full second opinion protocol (a-c), 282 (66.4%) were not recommended surgery by the second opinion (b + d + e) and 143 (33.6%) were recommended spine surgery (f)

The procedure for all included participants is shown in Fig. 1. All participants initially met with a senior nurse who explained how the second opinion process worked. She recorded the community spinal surgeon’s diagnosis, collected demographic data and asked each participant to complete the following measures:

1. Overall pain over the last week, measured on a 0 to 10 visual analogue scale (VAS) (with 0 indicating no pain, 10 the maximum pain) [15].
2. The Short Form-36 (SF-36), a tool with nine dimensions covering physical and psychological aspects of function and quality-of-life [16]. Each of the dimensions is transformed to a score ranging from 0 to 100, with higher scores indicating better health and functioning.

Participants with low back pain were also asked to complete:

1. The Roland–Morris Disability Questionnaire (RMDQ), a disability index in which scores range from 0 to 24, with higher numbers indicating worse physical functioning [17, 18].
2. The Oswestry Disability-Index (ODI), another disability index, which includes 10 six-point scales [19, 20]. The sum of the scale scores is expressed as a percentage of the maximum scores, with the total score ranging from 0 (no disability) to 100 (maximum disability).

Each participant attended two medical appointments with clinicians trained in evidence-based management of back and neck pain, the first with a physiatrist and the second with a general orthopaedic surgeon (who does not perform spine surgery). Both clinicians were blinded to the diagnosis and surgical treatment recommendation proposed by the first opinion. Each clinician performed a clinical assessment and reviewed any relevant investigations (usually plain radiographs, magnetic resonance imaging and electroneuromyography), and where considered necessary, requested new investigations.

Following their independent reviews, the physiatrist and orthopaedic surgeon compared diagnoses and recommended treatment for each participant, and, if these differed, sought to achieve consensus by discussion. When there was consensus that surgery was indicated, or when consensus could not be reached, participants were referred to a spinal review board. The board comprised nine senior spine surgeons (three orthopaedic surgeons and six neurosurgeons), each of whom had more than 15 years of spinal surgery experience. All are self-employed and not employed by the hospital. The spine review board made a final diagnosis and treatment recommendation.

### Treatment at HIAE

All participants who received a second opinion recommending conservative management (CM) or surgery were offered treatment at HIAE. Surgery at HIAE was performed by one of the nine surgeons on the spine review board, chosen at randomly.

CM treatment took place in the rehabilitation centre and comprised 20 physiotherapy sessions of manual therapy and exercise focused upon spine stabilisation, and six sessions of acupuncture. Additionally medication (i.e. analgesia, non-steroidal anti-inflammatory drugs, muscle relaxants, glucocorticoids), TENs and activities and job modifications were also offered if considered appropriate. All participants who received CM were reviewed after 10 physiotherapy appointments. If their progress was satisfactory they continued CM. If they had not improved, they were referred to a spine surgeon from the program.

Outcomes (overall neck or low back pain and SF-36, plus the RMDQ and ODI for those with low back pain), were collected by telephone at 1, 3, 6 and 12-months following treatment in all participants treated at HIAE. Adverse events were also collected at each time point with the use of open-ended questions.

### Data analysis

Descriptive analyses (*N*, %) were performed comparing the first and second opinion diagnoses and management plans. We compared the first opinion diagnosis (community spinal surgeon) with the diagnosis made by the HIAE physiatrist and orthopaedic surgeon. We compared the treatment recommendations of the community spinal surgeon with the final second opinion (physiatrist and orthopaedic surgeon review and/or spine board review as applicable). For participants who declined consultation with the spinal board, the diagnosis recorded after the physiatrist and orthopaedic surgeon consensus meeting was recorded as the final diagnosis, but no final treatment plan was recorded.

A wide variety of diagnostic labels were used and many different labels were used for the same condition. For clarity we categorised diagnoses post-hoc into the following categories: cervical radiculopathy (included ‘cervical disc herniation’ and ‘cervical discopathy’), cervical myelopathy, neck pain (included ‘cervical zygapophyseal pain’, ‘cervical osteoarthritis’, ‘cervical spondylosis’ and ‘mechanical neck pain’), lumbar radiculopathy (included ‘lumbar disc herniation’ and ‘lumbar discopathy’), lumbar canal stenosis, low back pain (included ‘lumbar zygapophyseal pain’, ‘lumbar osteoarthritis’, ‘lumbar spondylosis’ ‘mechanical low back pain’, ‘lumbar instability’, ‘spondylodisciitis’ and ‘spondylolisthesis’), failure of previous spine surgery (included ‘failed back’, ‘mechanical implant failure of lumbar fusion’, ‘non-union of cervical fusion’, ‘non-union of lumbar fusion’, ‘complication of disc arthroplasty’ and ‘complication of intraspinous spacer’) and non-spinal diagnoses. We also separated cervical and lumbar radiculopathy and failed spinal surgery if the second opinion indicated a different level.

Baseline patient-reported data were summarised by final opinion outcome (CM, surgery, non-spine condition (so neither CM nor surgery), or refused spine board consultation) and by site of condition (cervical, lumbar, or non-spine). The *p*-values for differences between groups were obtained from linear regression of the groups on the relevant outcome.

Baseline and outcome data of patients who elected to be treated at HIAE were summarised by study group (patients treated conservatively or surgically) using descriptive statistics. Patients in the CM group who were reported to have failed treatment and were referred for surgical intervention were analysed with the CM group.

Baseline comparability was assessed with t-tests. The mean differences in outcome change scores by management and location were obtained from linear regression of 12-month outcomes adjusted for baseline values of the outcome. A *p*-value < 0.05 was considered statistically significant. For our primary outcome of pain we used the accepted minimal clinically important difference for pain (VAS of 1.5 units on an 11-point scale (0 to 10)) [15, 21].

## Results

A total of 544 patients were referred for a second opinion during the recruitment phase of our study (Fig. 1). Of these, 16 (2.9%) did not meet the study inclusion criteria, 43 (7.9%) refused participation and 485 were included in the study. There were no sex differences between those who participated and those who refused participation (i.e. proportions of males refusing (46.5%) or participating (46.2%)). The mean age (standard deviation) of participants was 43.9 (11.3) years. We have no further information about those who refused study participation.

### Comparison of first and second opinions: Diagnoses

**Fig. 2:**
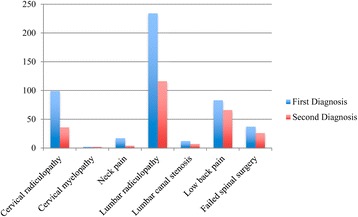
Concordance of diagnoses made by the spinal surgeon (first diagnosis) and the HIAE physiatrist and orthopaedic surgeon (second diagnosis), *N* = 484*. *Excludes one participant where a diagnosis was not recorded by the spinal surgeon

Table 1 displays the diagnoses made by the initial surgeon compared with the consensus diagnoses of the physiatrist and orthopaedic surgeon and Fig. 2 displays the data graphically. The diagnosis was concordant in only 257 (53.0%) participants and 87 (17.9%) participants received a non-spinal second diagnosis. The second opinion concurred with a diagnosis of cervical radiculopathy in 36/99 (36.4%) participants while the remaining participants were considered to have either a different level radiculopathy (*N* = 8), neck pain (*N* = 14) or a non-spinal diagnosis (*N* = 41), most commonly myofascial pain syndrome (*N* = 27). The second opinion concurred with a diagnosis of lumbar radiculopathy in 116/234 (49.6%) participants, while alternate second opinion diagnoses included a different level radiculopathy (*N* = 18), low back pain (*N* = 75), non-spinal diagnoses (*N* = 20), thoracic pain (*N* = 2), lumbar canal stenosis (*N* = 1) and coccygeal pain (*N* = 1).

**Table 1:** Comparison of the diagnoses made by the community spinal surgeons compared with the HIAE physiatrist and orthopaedic surgeon, agreement shown in bold (*N* = 485)

Diagnosis of the community spinal surgeon									
Diagnosis of the HIAE physiatrist and orthopaedic surgeon	Cervical radiculopathy^a^	Cervical myelopathy	Neck pain^b^	Lumbar radiculopathy^c^	Lumbar canal stenosis	Low back pain^d^	Failed spine surgery^e^	Not reported	Total
Cervical radiculopathy^a^	**36**		2	1					39
Cervical radiculopathy, different level	8								8
Cervical myelopathy		**2**							2
Neck pain^b^	14		**4**						18
Lumbar radiculopathy^c^				**116**		2			118
Lumbar radiculopathy, different level				18					18
Lumbar canal stenosis				1	**7**				8
Low back pain^d^				75	4	**66**	7	1	153
Thoracic pain			1	2					3
Coccyx pain				1					1
Failed spine surgery^e^							**26**		26
Failed spine surgery, different level			1		1	2			4
Non-spinal condition^f^	41		9	20		13	4		87
Total	99	2	17	234	12	83	37	1	485

^a^Includes cervical disc herniation and cervical discopathy; ^b^Includes cervical zygapophyseal pain, cervical osteoarthritis, cervical spondylosis and mechanical neck pain; ^c^Includes lumbar disc herniation and lumbar discopathy; ^d^Includes lumbar zygapophyseal pain, lumbar osteoarthritis, lumbar spondylosis’, ‘mechanical low back pain’, ‘lumbar instability’, ‘spondylodisciitis’ and ‘spondylolisthesis’; ^e^Includes all procedures that need another surgical intervention (failed back, mechanical implant failure of lumbar fusion, non-union of cervical fusion, non-union of lumbar fusion, complication of disc arthroplasty, complication of intraspinous spacer)

^f^First opinion of cervical radiculopathy was diagnosed by the second opinion as myofascial pain syndrome (*N* = 27), carpal tunnel syndrome (*N* = 5) shoulder impingement (*N* = 3), headache (*N* = 2), calcific tendinitis (*N* = 1), dizziness (*N* = 1), diabetic polyneuropathy *N* = 1) and cubital tunnel syndrome (*N* = 1); first opinion of neck pain was diagnosed by the second opinion as myofascial syndrome (*N* = 8) and shoulder impingement (*N* = 1); first opinion of lumbar radiculopathy was diagnosed by the second opinion as myofascial syndrome (*N* = 14), peripheral neuropathy (*N* = 1), medication induced polyneuropathy (*N* = 1), post-polio syndrome (*N* = 1), trochanteric bursitis (*N* = 1), meralgia paraesthetica (*N* = 1) and rheumatoid arthritis (*N* = 1); first opinion of lumbar instability was diagnosed by the second opinion as myofascial syndrome (*N* = 1); first opinion of low back pain was diagnosed by the second opinion as myofascial syndrome (*N* = 7), trochanteric bursitis (*N* = 2), vascular claudication (*N* = 1), sacroiliitis (*N* = 1) and rheumatoid arthritis (*N* = 1); and first opinion of failed spine surgery was diagnosed by the second opinion as myofascial syndrome (*N* = 2). Osteoarthritis hip (*N* = 1) and reflex sympathetic dystrophy (*N* = 1)

### Comparison of first and second opinions: Recommended treatment

Based upon the assessment of the physiatrist and orthopaedic surgeon, CM was recommended for 168 (34.6%) participants, 290 (59.8%) were referred to the spine surgeon review board, and 27 (5.6%) were recommended neither. Of the 290 participants offered spine board review, 60 (12.4%) participants did not attend. We have no further follow up for these participants. For the 230 who attended the spine board review, surgery was recommended for 143, CM for 67 and another 20 were recommended neither. Overall, among the 425 participants who completed the full second opinion protocol, only 143 (33.6%) participants were recommended to have surgery by the second opinion (Fig. 1).

**Fig. 3:**
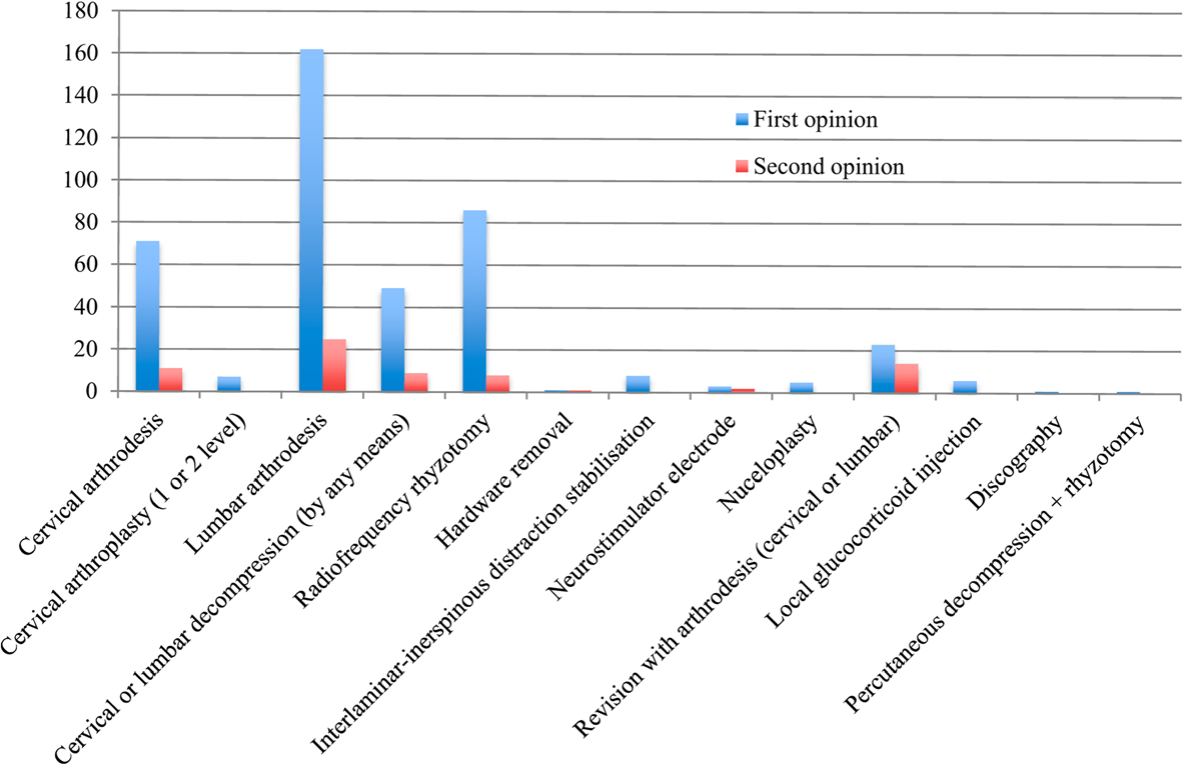
Concordance of treatment recommendations made by the spinal surgeon (first opinion) and the HIAE physiatrist and orthopaedic surgeon (second opinion), *N* = 424*. *Excludes 60 participants that did not attend the spine review board and one participant where a treatment recommendation was not recorded by the spinal surgeon

Table 2 compares the treatment recommendations of the community spinal surgeon compared to the final second opinion (physiatrist and orthopaedic surgeon review and/or spine board review as applicable) and Fig. 3 displays the data graphically. There was a range of operations recommended for the 60 patients who were referred to the spine board but did not attend (first row, Table 2). For the remainder, the same specific treatment was recommended in only 66/425 (15.5%) participants. CM rather than surgery was the final recommendation for 235 (55.3%) participants, different surgical treatments were recommended for 77 (18.1%), while 47 (11.1%) participants were offered neither CM nor surgery as they were not considered to have a spinal condition.

**Table 2:** Comparison of the recommended treatment of the community spinal surgeons compared with the HIAE physiatrist and orthopaedic surgeon and/or spinal review board, agreement shown in bold (*N* = 485)

Recommended treatment of the community spinal surgeon																			
Recommended treatment of the HIAE physiatrist and orthopaedic surgeon and/or spine review board	Cervical arthrodesis	Cervical disc arthroplasty (1 level)	Cervical disc arthroplasty (2 levels)	Lumbar arthrodesis	Cervical or lumbar decompression	Endoscopic lumbar decompression	Percutaneous decompression	Percutaneous decompression +rhyzotomy	Radiofrequency rhyzotomy	Hardware removal	Interlaminar- interspinous distraction stabilisation	Neuro-stimulator electrode	Nucleoplasty	Revision with arthrodesis (cervical)	Revision with arthrodesis (lumbar)	Local glucocorticoid and local anaesthetic injection^a^	Discography	Not reported	Total
Refused spine review board attendance	8	1		34	1		2		3	1				1	8		1		60
Cervical arthrodesis	**11**						1		1		1								14
Cervical arthrodesis, different level	6																		6
Cervical disc arthroplasty																			0
Cervical disc arthroplasty (2 levels)																			0
Lumbar arthrodesis				**25**	1						1								27
Lumbar arthrodesis, different level				11															11
Decompression	5			30	**7**		2		1		2							1	48
Percutaneous decompression with rhyzotomy																			0
Radiofrequency rhyzotomy									**8**										8
Hardware removal										**1**									1
Neuro-stimulator electrode												2							2
Revision with arthrodesis (Cervical)									1										1
Revision with arthrodesis (Lumbar)									2						**14**				16
Coccyx resection				1															1
Local glucocorticoid and local anaesthetic injection^a^				4					4										8
Conservative treatment	35	3	2	86	16	1	14	1	56		4	1	4		6	5		1	235
Non-spine condition	14	1	1	5	4		3		13				1	1	2	1	1		47
Total	79	5	3	196	29	1	22	1	89	2	8	3	5	2	30	6	2	2	485

^a^Considered ‘surgery’ because procedure performed under anaesthesia in the operating room

### Comparison of participants according to treatment recommended at HIAE

Table 3 displays the baseline characteristics and outcome data of study participants according to whether the final treatment recommendation was surgery (*N* = 143) or CM (*N* = 235). Compared with participants recommended for CM, those allocated to surgery were older, had higher pain scores and poorer SF-36 bodily pain and SF-36 role physical scores. Those with low back complaints recommended for surgery also had significantly worse RMDQ and ODI scores.

**Table 3:** Comparison of demographic and baseline outcome data by recommended final treatment for all participants and for those treated at HIAE and adjusted mean differences in change scores for those treated at HIAE

	All participants			Participants treated at HIAE									
	Surgery (*N* = 143)^b^	CM (*N* = 235)^a^		Surgery (*N* = 67)		CM (*N* = 99)			Surgery (*N* = 67)		CM (*N* = 99)		
			*p*-value^c^	Baseline				*p*-value^c^	12 months				Adjusted mean difference between groups (95% CI)^d^
				*N*		*N*			*N*		*N*		
Female, *N* (%)	75 (52)	120 (51)	0.8	67	30 (45)	99	47 (47)	0.7					
	Mean (SD)	Mean (SD)			Mean (SD)		Mean (SD)			Mean (SD)		Mean (SD)	
Age, years	45.2 (12.6)	42.3 (10.7)	0.02	67	44.3 (10.6)	99	46.8 (14.5)	0.2					
Pain (0 to 10)^e^	7.8 (2.2)	7.3 (2.1)	0.03	67	7.6 (2.4)	99	7.0 (2.2)	0.1	57	3.1 (3.0)	77	3.4 (3.3)	0.53 (−0.53 to 1.60)
SF 36 (0–100)^f^													
VT	38.5 (23.5)	38.5 (21.2)	0.99	67	41.6 (26.5)	98	39.4 (21.5)	0.6	54	63.3 (23.4)	79	61.5 (24.3)	−1.6 (−9.1 to 5.9)
PF	32.9 (17.5)	35.3 (18.4)	0.2	66	38.6 (19.5)	98	38.9 (19.5)	0.9	54	64.4 (23.7)	79	62.2 (25.6)	−3.3 (−11.4 to 4.7)
BP	22.8 (18.1)	28.2 (18.3)	0.006	67	25.7 (20.8)	98	30.2 (19.9)	0.2	54	63.4 (27.5)	79	58.2 (30.5)	−7.3 (−17.4 to 2.9)
GH	55.5 (23.5)	55.1 (22.1)	0.9	66	59.2 (23.43)	99	57.2 (21.5)	0.6	54	76.6 (23.1)	79	69.6 (24.0)	−6.6 (−14.3 to 1.1)
RP	18.3 (26.4)	28.4 (32.6)	0.002	67	10.8 (26.5)	98	22.2 (37.1)	0.03	54	59.3 (46.4)	79	56.3 (46.9)	−8.1 (−24.4 to 8.2)
RE	28.4 (40.0)	36.7 (43.4)	0.1	67	27.4 (40.2)	98	34.1 (43.8)	0.2	54	80.9 (38.6)	79	73.8 (42.9)	−9.4 (−23.7 to 5.0)
SF	22.8 (28.6)	27.5 (31.8)	0.2	67	38.4 (27.9)	98	45.7 (27.5)	0.1	54	73.6 (29.3)	79	70.3 (32.6)	−5.0 (−15.7 to 5.8)
MH	48.8 (22.9)	49.5 (22.4)	0.8	67	50.7 (24.5)	98	48.4 (23.4)	0.5	54	73.8 (23.4)	79	69.2 (27.1)	−4.1 (−12.4 to 4.2)
HT	30.9 (25.9)	31.1 (23.5)	0.9	67	27.6 (25.4)	98	34.2 (24.2)	0.1	54	75.5 (26.8)	79	70.6 (27.4)	−4.9 (−14.5 to 4.7)
RMDQ (0–24)^e^	17.3 (4.8)	13.5 (5.8)	<0.001	56	18.1 (4.4)	75	12.3 (5.4)	<0.001	48	8.8 (7.5)	61	8.3 (6.8)	1.5 (−1.6 to 4.7)
ODI (0–100)^e^	50.1 (16.9)	42.2 (16.3)	<0.001	56	47.8 (17.4)	75	42.8 (16.0)	0.1	48	28.1 (18.9)	60	27.4 (19.3)	0.1 (−7.1 to 7.3)

^a^Ns for outcome variables range from 170 to 221; ^b^Ns for outcome variables range from 112 to 135; ^c^ *p*-values from linear regression; ^d^Adjusted for baseline score of outcome variable; ^e^Higher scores indicate greater pain or disability; ^f^Higher scores indicate better quality of life or function

### Outcome of treatment at HIAE

One hundred and sixty-six participants agreed to be treated at HIAE. Sixty-seven were treated with surgery (56 low back, 11 neck) and 99 were treated with CM (75 low back, 24 neck). At baseline, participants in the surgery group had poorer SF-36 RP (role physical) and RMDQ scores than those in the CM group but they did not differ for other outcomes (Table 3).

**Fig. 4:**
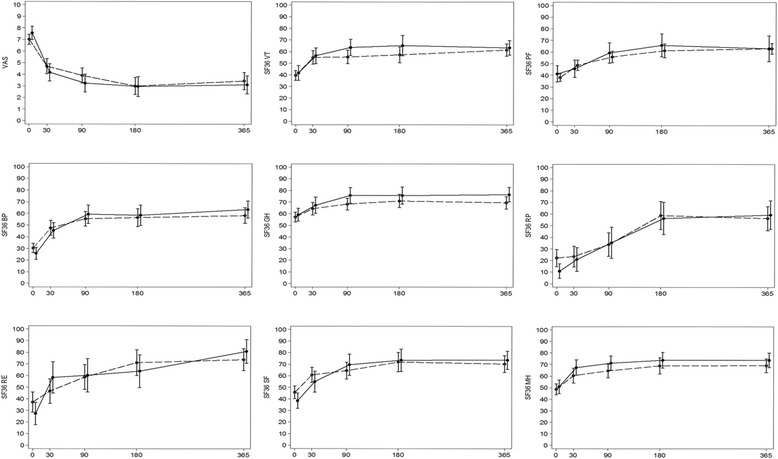
Mean outcome scores and 95% confidence intervals for the mean at each follow-up time point by treatment. Solid lines indicate Surgery; dotted lines indicate Conservative management

Complete 12-month follow up data were available for 56 (83.6%) participants treated with surgery and 77 (77.8%) participants treated with CM. The health insurance company reported that none of the participants treated with CM who failed to attend the 12-month follow-up received spine surgery during the 12-month period after the second opinion. Among participants with 12-month follow up, there was no significant between-group difference on any measured outcome (Table 3 and Fig. 4). Both treatment groups improved over time. Post-hoc analysis requested by a manuscript reviewer found that 46 patients (80.7%) in the surgery group and 50 (64.9%) in the CM group showed a reduction in pain VAS greater than 1.5 units (χ^2^ *p*-value = 0.045). Among participants treated for a neck complaint, one participant in the surgical group was reported to have failed treatment. No adverse events were reported in either group. Among participants treated for a low back complaint, four participants in the surgical group were reported to have failed treatment and were referred for a different surgical intervention, while nine participants in the CM group were reported to have failed treatment and were referred for surgical intervention. Three adverse events were reported in the surgery group (superficial infection in two participants and one participant had a bowel obstruction) and no adverse events were reported in the CM group.

## Discussion

We found significant discrepancies in the diagnoses and treatment recommendations between spine surgeons in private practice and multidisciplinary second opinions obtained at a non-for-profit private hospital for 485 patients with back and neck complaints initially offered spine surgery. Among those who completed the full second opinion protocol, two-thirds did not receive a similar opinion; the diagnosis differed in over a half and almost 10% were not considered to have a spinal disorder. In only 15% of cases was there full agreement between the first final opinions with respect to the specific surgical intervention required. Cervical and lumbar radiculopathy were the most common cervical and lumbar diagnoses provided by the first opinion surgeons that were disputed by the second opinion. The majority were reclassified as myofascial pain syndrome and mechanical low back pain respectively.

Our data are consistent with previous studies that have found that up to 61% of recommendations for surgical interventions for back or neck pain may be considered discordant with a second opinion [22–26]. They are also in keeping with a recent paper from a private spinal surgery group practice in Brazil [27]. In this study 94 patients received a second opinion over a one-year period. There was complete treatment concordance between first and second opinions for 22 patients (23%), partial concordance for 28 patients (30%), and discordance for 44 patients (47%). For lumbar disease, the second opinion resulted in a 50% reduction in surgical procedures and 50% reduction in the rate of instrumentation.

In keeping with our data, a recent study demonstrated that consultation with a non-spine surgeon for patients offered elective spine surgery decreased the rate of spine surgery and patients were mostly satisfied with the results of their treatment [28]. While we cannot know for certain which of the two opinions was more ‘correct’ in our study, our findings support the hypothesis that a second opinion may reduce potentially unnecessary spine surgery without resulting in poorer outcomes. In the subset of patients treated at HIAE we observed comparable outcomes among participants treated with CM compared to those treated surgically, even after adjusting for baseline differences of greater disability among those offered surgery. While this may suggest that the HIAE triage process was effective in correctly assigning patients to the most appropriate care, it is possible that some of the patients treated with surgery would have also improved with CM and vice versa. Further definitive conclusions cannot be drawn due to the lack of follow up for patients treated outside of the HIAE.

Our study has also highlighted the lack of uniformity in diagnostic labels for back and neck complaints and the wide variety of labels used even for the same putative cause. While we did not collect any data about how diagnoses were made, there is consistent evidence that it is not possible to attribute a specific cause for over 85% of patients with non-specific low back pain, and at least the same proportion of patients with non-specific neck pain. Most physical examination tests for people with back pain perform poorly in distinguishing between those with and without specific causes for their problem [29–31]. Similarly there is poor correlation between clinical features and imaging findings [32]. These issues are likely to contribute to wide practice variation and unwarranted spine surgery rates. Other explanations for disparate diagnoses between community spinal surgeons and the HIAE team include differences in education and training and/or community and academic standards. It is also possible that financial incentives inherent in surgical practice in the private sector played a role.

The strengths of our study are that it was performed in a real world setting and is therefore likely to be generalizable, at least to other settings in Brazil. We found that obtaining a second opinion among those with private health insurance (albeit a minority of the general population), was both feasible and acceptable to most patients. Clinicians at HIAE were blinded to the diagnoses and treatment recommendations given by the community spinal surgeon, reducing the potential for bias.

There are also important limitations of our study. Firstly, the second opinion physicians (physiatrists and orthopaedic surgeons) are salaried employees of HIAE and therefore may have an inherent bias towards justifying their importance by disputing private practice diagnoses and by saving HIAE money recommending against surgery. However, we think this is unlikely to account for the large observed discrepancy in diagnoses and treatment recommendations between first and second opinions. We did not record the time interval between the first and second opinions and it is therefore possible that for some patients, differences of opinion regarding surgery may be explained by improvement in clinical status over time. Patients who were referred to HIAE had an existing relationship with their original spine surgeon and more than half chose not to have treatment at HIAE. We were unable to obtain follow up data for these patients and it is therefore possible that their outcomes differed systematically from patients who chose to have treatment at HIAE. We did not collect data regarding the duration of symptoms or concurrent management and these might be important confounders.

In addition, we did not record the initial diagnosis and management recommendations of the physiatrists and orthopaedic surgeons so do not know their level of concordance. Unfortunately we also did not collect sufficient data to be able to report the degree of concordance in diagnosis between the second opinion physiatrist and orthopaedic surgeon compared with the spinal review board. Finally only participants deemed to require surgery or for whom consensus could not be reached were reviewed by the spine review board and it is therefore possible, but in our view unlikely, that some patients recommended CM by the physiatrist and orthopaedic surgeon could have been recommended surgery by the spine board.

With rising medical costs in in Brazil, queries about unnecessary treatment have become more frequent. In our setting healthcare companies are spending billions on unnecessary surgery and other medical procedures. The lack of effective health insurance for the majority of population and the absence of laws that rule malpractice (which often leads to over-treatment) are some of the issues Brazilian people face, particularly those who suffer from spine complaints. This may be ameliorated in part by mandating a second opinion when surgery is offered to all patients with a degenerative spinal condition. While this could be sought from another spinal surgeon, a second opinion provided by centres of excellence that provide multidisciplinary assessment and management could optimise quality improvement among health care providers, reduce costs through high volume efficiencies and create market differentiation with high patient satisfaction [33].

Although there are numerous and consistent clinical guidelines for the management of back pain [11–13, 34], there continues to be large unwarranted variations in clinical practice in this field [29–32]. Further efforts are needed to ensure that clinical practice is in line with ‘doing what is right for patients’ and represent good value for money [35, 36]. Our study provides evidence that requiring a second opinion reduces potentially unnecessary spine surgery in our setting and these findings are likely to be generalisable.

## Conclusions

In summary, we have demonstrated that provision of a second opinion to people who have been recommended spinal surgery in our setting significantly reduced the proportion who underwent surgery. In a subset of patients treated at HIAE according to the second opinion we observed comparable outcomes among participants treated with CM compared to those treated surgically suggesting that at the very least it does not cause harm. We recommend that Brazilians and others contemplating spine surgery consider seeking a second opinion.

## Abbreviations

CM: Conservative management
HIAE: Hospital Israelita Albert Einstein
ODI: The Oswestry Disability-Index
RMDQ: The Roland–Morris Disability Questionnaire
SF-36: The Short Form-36
TENs: Transcutaneous Electrical Nerve stimulation
VAS: Visual analogue scale
